# Reproductive desire in women with HIV infection in Spain, associated factors and motivations: a mixed-method study

**DOI:** 10.1186/1471-2393-14-194

**Published:** 2014-06-05

**Authors:** Victoria Hernando, Belén Alejos, Débora Álvarez, Marta Montero, Mª Jesús Pérez-Elías, Jose Ramón Blanco, Mar Masiá, Jorge del Romero, Ignacio de los Santos, Isabel Rio, Alicia Llácer

**Affiliations:** 1 Red de Investigación en Sida, Centro Nacional de Epidemiología, Instituto de Salud Carlos III, Avda. Monforte de Lemos, 5 28029, Madrid, Spain; 2 CIBER de Epidemiología y Salud Pública (CIBERESP), Madrid, Spain; 3 Hospital Universitario La Fe, Valencia, Spain; 4 Hospital Universitario Ramón y Cajal, Madrid, Spain; 5 Hospital Universitario San Pedro-CIBIR, Logroño, Spain; 6 Hospital Universitario de Elche, Alicante, Spain; 7 Centro Sanitario Sandoval, Madrid, Spain; 8 Hospital Universitario La Princesa, Madrid, Spain

**Keywords:** Reproductive desire, HIV infection, Women, Disclosure, Social support

## Abstract

**Background:**

Antiretroviral therapy has created new expectations in the possibilities of procreation for persons living with HIV. Our objectives were to evaluate reproductive desire and to analyze the associated sociodemographic and clinical factors in HIV-infected women in the Spanish AIDS Research Network Cohort (CoRIS).

**Methods:**

A mixed qualitative-quantitative approach was designed. Women of reproductive age (18–45) included in CoRIS were interviewed by phone, and data were collected between November 2010 and June 2012 using a specifically designed questionnaire. Reproductive desire was defined as having a desire to be pregnant at present or having unprotected sex with the purpose of having children or wanting to have children in the near future.

**Results:**

Overall, 134 women were interviewed. Median age was 36 years (IQR 31–41), 55% were Spanish, and 35% were unemployed. 84% had been infected with HIV through unprotected sex, with a median time since diagnosis of 4.5 years (IQR 2.9-6.9). Reproductive desire was found in 49% of women and was associated with: 1) Age (women under 30 had higher reproductive desire than those aged 30–39; OR = 4.5, 95% CI 1.4-14.3); 2) having no children vs. already having children (OR = 3.2; 1.3-7.7 3); Being an immigrant (OR = 2.2; 1.0-5.0); and 4) Not receiving antiretroviral treatment (OR = 3.6; 1.1-12.1). The main reasons for wanting children were related to liking children and wanting to form a family. Reasons for not having children were HIV infection, older age and having children already. Half of the women had sought or received information about how to have a safe pregnancy, 87% had disclosed their serostatus to their family circle, and 39% reported having experienced discrimination due to HIV infection.

**Conclusions:**

The HIV-infected women interviewed in CoRIS have a high desire for children, and the factors associated with this desire are not fundamentally different from those of women in the general population. Maternity may even help them face a situation they still consider stigmatized and prefer not to disclose. Health-care protocols for handling HIV-positive women should incorporate specific interventions on sexual and reproductive health to help them fulfill their procreation desire and experience safe pregnancies.

## Background

The introduction of combined antiretroviral treatment (cART) has drastically reduced HIV-related mortality and morbidity, and has yielded improvements in the quality of life and prognosis of people living with HIV [1]. Antiretroviral treatment, together with programmed caesarean sections, have significantly reduced mother-to-child transmission to rates under 2% [2]. All this has led a growing number of HIV-infected persons to consider the possibility of having children. Studies in women diagnosed with HIV infection in Africa, Europe and North American show that many of these women decide to have children even after their HIV diagnosis [3-5]. However, to our knowledge, there are no studies assessing the reproductive desires of HIV-infected women in Spain.

A large variety of individual, clinical and social factors influence reproductive desire in HIV-infected persons. Some studies show that HIV infection is not a dominant factor in the desire to have children, and that the factors influencing this desire are similar in both HIV-infected women and those in the general population [3,4]. HIV-infected women of reproductive age have reported high anxiety related to reproductive concerns, such as a great desire for pregnancy along with experiences of stigmatization for trying to become pregnant [6,7].

In Spain, women comprised 17% of new HIV infections and 21% of AIDS cases diagnosed in 2011. Most of them acquired their infection through sexual contact (83% and 65% of HIV and AIDS cases, respectively) [8].

The objectives of this work were to evaluate the reproductive desires of HIV-positive women included in the CoRIS cohort, to analyze the different motivations and to identify the associated factors. Such knowledge will be important for developing programs to support these women in planning safe pregnancies.

## Methods

### Design, sample frame and subjects

To achieve the study objectives, we designed a cross-sectional observational study framed within the cohort of HIV-infected adults in the AIDS Research Network (CoRIS). CoRIS is a prospective, open and multicenter cohort of patients newly diagnosed with HIV who are over 13 years of age and naïve to antiretroviral treatment at cohort entry [9,10]. Of the 28 centers participating in CoRIS, nine hospitals and one sexually transmitted disease clinic in each of eight cities (Madrid, Valencia, Logroño, Santa Cruz de Tenerife, Elche, Alicante, Murcia, and Donostia) from six Autonomous Communities of Spain participated in this project (see Additional file 1). Each patient signed an informed consent form, and the study was approved by the Ethics Committee of the Carlos III Institute of Health.

The first phase of the study included all women in the CoRIS cohort in the participating hospitals and centers in January 2010 who were between 18 and 49 years of age. Healthcare and/or clinical staff in each participating center contacted the women selected to explain the project and to request their participation and written informed consent. After receiving their consent, in the second phase a person from the research team contacted each woman by telephone to conduct the interview. Of the 494 women selected, 240 (48%) could not be located or were no longer being followed up in the hospital or healthcare center. Of the remaining 254, 155 (61%) agreed to participate, 63 (25%) refused, and 36 (14%) were excluded for various reasons (not speaking Spanish well, not having a telephone, living in the street, or being in prison). One of the hospitals included in the study began to recruit women when the field work was almost completed, therefore the number of women included was very small (n = 6). A total of 161 women were finally interviewed. The interviews were conducted between November 2011 and December 2012.

The main outcome of this analysis was *reproductive desire,* defined as having a desire to be pregnant at present OR having unprotected sex with the purpose of having children OR wanting to have children in the near future. Thus, the sample was limited to women of reproductive age (18–45 years), a total of 134 women.

### Definition of variables

A questionnaire specific to this study was designed and validated in a pilot test with 10 women. The final questionnaire included a mixed qualitative-quantitative approach (see Additional file 2). Quantitative data were collected on sociodemographic, epidemiological and clinical variables, as well as sexual and reproductive history. To collect information on the reasons underlying reproductive desire, issues pertaining to reproductive health and disclosure of HIV infection, we chose a qualitative approach using open-ended questions. Two experienced interviewers conducted the interviews by telephone.

*Country of origin* was categorized as “Spain” or “Other countries”. Women from other countries were subsequently categorized according to their *region of origin* into three groups: “Europe”, “Latin America” and “Africa”. *Educational level* was defined as “low” if women had no formal education or primary education, “medium” if women had completed secondary education, and “high” if women had a university degree. *Current* o*ccupational status* was defined as “employed”, “unemployed”, or “other” – which included students, homemakers and pensioners. *Current partner and cohabitation status* was grouped into “has a partner and they live together”, “has a stable partner but they do not live together” and “no stable partner”. *HIV transmission category* was classified as “injecting drug user” (IDU), “heterosexual contact” or “other”. *Hepatitis C Virus (HCV) serological status* was classified as positive or negative antibodies, *AIDS defining conditions* as “Yes” or “No”, and *Time since HIV diagnosis* as “<3”, “3-6” and “>6” years.

In accordance with the previous answers, women were then asked the open-ended question “Why do you want to have children in the near future?” or “Why do you not want to have children/more children in the near future?”. Answers were recorded and later grouped into the following categories for those who “Desired to have children”: (1) To have a normal life, (2) To form a family, (3) They like children and want to have them, (4) To experience maternity, (5) To have someone to take care of them in the future, (6) Their partners want to have children, and (7) Religious beliefs. The categories for those who did “Not wish to have children” were: (1) Age (too old to have children), (2) They already have children, (3) HIV infection, (4) Personal reasons, (5) Economic reasons, (6) Drug abuse, (7) Do not like or want to have children, (8) Health problems other than HIV infection.

Women were also asked if they had looked for or received information about sexual and reproductive health, and whether they had disclosed their HIV infection to their closest relatives and friends. Social support was evaluated through a 4-item MOS-SSS version [11], each representing a social dimension: (1) Someone to help you with daily chores if you were sick [*Tangible*], (2) Someone to turn to for suggestions about how to deal with a personal problem [*Emotional/Informational*]; (3) Someone to do something enjoyable with [*Positive social interaction*] and (4) Someone to love and make you feel wanted [*Affectionate*]. Each item ranged from 1 (never) to 5 (always), so a higher score meant better social support. An overall score was calculated and transformed into a 0–100 scale using the formula: 100 × [(observed score sum – minimum possible score)/(maximum possible score – minimum possible score)].

### Statistical analysis

Women’s characteristics were described according to reproductive desire using frequency tables for categorical variables and median and interquartile range (IQR) for continuous ones. The chi-squared test for independence was used for comparison of categorical variables and the non-parametric Kruskal-Wallis test for continuous variables.

Multivariate logistic regression models were fitted to evaluate the association of women’s characteristics with reproductive desire. Crude and adjusted odds ratios (ORs) with their 95% confidence intervals (95% CI) were obtained as the measure of association. Predictive modeling was performed, and variables with a P-value lower than 0.05 were retained in the multivariable model.

All statistical analyses were performed using Stata software (Version 11.0, College Station, Texas).

## Results

### Description of women

A total of 134 women were interviewed, with a median age of 36 years (IQR: 31–41). Some 54% (n = 72) were of Spanish origin and, of the remaining 62, 44% (n = 27) were from Latin America, 32% (n = 20) from Europe, and 24% (n = 15) from Africa. The foreign women had been in Spain a median of 8 years (IQR: 6–11 years). About 77% (n = 103) of women had medium educational level and a similar proportion had low and high education: 11% (n = 15) and 12% (n = 16), respectively. With regard to employment status, 50% (n = 67) were currently employed, 35% (n = 47) were unemployed and 15% (n = 20) had other occupational status (homemakers, students or pensioners). A total of 67% (n = 90) had a stable partner at the moment and, of these, 77% (n = 69) lived with their husband or current partner.

At the time of the interview, 21% (n = 28) reported having a health-related circumstance which complicated or impeded procreation. Among them, 43% (n = 12) had undergone tubal ligation. Only 2% (n = 2) of their male partners had fertility problems.

Regarding their HIV infection, 51% (n = 69) had been diagnosed with HIV before 30 years of age, 37% (n = 50) between 30 and 39 years, and 11% (n = 15) at age 40 or over. The majority (84%, n = 112) had acquired the infection though heterosexual exposure and 13% (n = 17) through injection of drugs. About 26% (n = 35) had been diagnosed with HIV for less than 3 years, 39% (n = 52) for between 3 and 6 years, and 35% (n = 47) for more than 6 years. Most women (89%, n = 119) reported being asymptomatic, while only 7% (n = 9) were on AIDS stage. In all, 85% (n = 114) were on antiretroviral treatment at the time of the interview. HCV coinfection was present in 15% (n = 20) of women.

Table 1 shows the descriptive analysis of sociodemographic, reproductive and clinical characteristics of women included in the study, stratified by reproductive desire.

**Table 1:** Characteristics of the study population, by reproductive desire

		**Reproductive desire**		
	**Total**	**Yes**	**No**	**p**
	**n (%)**	**n (%)**	**n (%)**	
Women	134 (100)	66 (49)	68 (51)	
***Sociodemographic variables***				
Age, years				
<30	28 (20.9)	23 (34.8)	5 (7.4)	<0.001
30-39	61 (45.5)	32 (48.5)	29 (42.6)	
40-45	45 (33.6)	11 (16.7)	34 (50.0)	
Median (IQR)	36 (31–34)	32 (28–37)	39.5(35–42.5)	<0.001
Country of origin				
Spain	72 (53.7)	27 (40.9)	45 (66.2)	0.003
Others	62 (46.3)	39 (59.1)	23 (33.8)	
Region of origin (if not Spanish)				
Europe	20 (32.3)	11 (28.2)	9 (39.1)	0.091
Latin America	27 (43.6)	15 (38.5)	12 (52.2)	
Africa	15 (24.1)	13 (33.3)	2 (8.7)	
Length of stay in Spain, years				
Median (IQR)	8 (6–11)	7.5 (5–10)	11 (8–15)	0.021
Educational level				
Low	15 (11.2)	8 (12.1)	7 (10.3)	0.862
Medium	103 (76.9)	51 (77.3)	52 (76.5)	
High	16 (11.9)	7(10.6)	9 (13.2)	
Current occupational status				
Employed	67 (50.0)	33 (50.0)	34 (50.0)	0.614
Unemployed	47 (35.1)	25 (37.9)	22 (32.3)	
Others (Student, Homemaker, Pensioner)	20 (14.9)	7 (12.1)	12(17.7)	
Current partner and cohabitation status				
With a partner and living together	69 (51.5)	30 (45.4)	39 (57.4)	0.297
With a partner but not living together	21 (15.7)	13 (19.7)	8 (11.8)	
Without a partner	44 (32.8)	23 (34.8)	21 (30.9)	
***Reproductive variables***				
Total pregnancies				
None	18 (13.4)	10 (15.2)	8 (11.8)	0.883
1-2	74 (55.2)	37 (56.1)	37 (54.5)	
3-4	30 (22.4)	14 (21.2)	16 (23.5)	
> = 5	12 (9.0)	5 (7.6)	7 (10.5)	
Number of children				
None	43 (32.1)	30 (45.4)	13 (19.1)	0.002
1-2	80 (59.7)	34 (51.5)	46 (67.7)	
> = 3	11 (8.2)	2 (3.1)	9 (13.2)	
Number of abortions and miscarriages				
None	60 (44.8)	21 (31.8)	39 (57.4)	0.010
1-2	64 (47.8)	38 (57.6)	26 (38.2)	
> = 3	10 (7.5	7 (10.6)	3 (4.4)	
***Clinical variables***				
Age at diagnosis, years				
<30	69 (51.5)	47 (71.2)	22 (32.5)	<0.001
30-39	50 (37.3)	15 (22.7)	35 (51.5)	
40-45	15 (11.2)	4 (6.1)	11 (16.2)	
Route of HIV transmission				
Injecting drug users (UDI)	17 (12.7)	5 (7.6)	12 (17.6)	0.182
Heterosexuals	112 (83.6)	59 (89.4)	53 (77.9)	
Others	5 (3.7)	2 (3.0)	3 (4.4)	
Current antiretroviral treatment				
Yes	114 (85.1)	52 (78.8)	62 (91.2)	0.044
No	20 (14.9)	14 (21.2)	6 (8.8)	
Time since HIV diagnosis, years				
<3	35 (26.1)	20 (30.3)	15 (22.1)	0.422
3-6	52 (38.8)	26 (39.4)	26 (38.2)	
>6	47 (35.1)	20 (30.3)	27 (39.7)	
HCV coinfection				
Yes	20 (14.9)	5 (7.6)	15 (22.1)	0.019
No	114(85.1)	61 (92.4)	53 (77.9)	
AIDS stage				
Yes	9 (6.7)	6 (9.1)	3 (4.4)	0.279
No	125 (93.3)	60 (90.9)	65 (95.6)	

### Reproductive history

Out of the 134 women included in the study, 116 (86.6%) had been pregnant in their lifetime and a total of 280 pregnancies were reported, 5 of them in process at the time of the interview (3.7%). Over half of the women (55%, n = 74) had been pregnant once or twice, 22% (n = 30) three or four times, and 9% (n = 12) more than four times. About 60% (n = 80) had one or two children, 8% (n = 11) had three or more children, and the rest (32%) did not have any children (Table 1).

Among the 116 women who had been pregnant, 54 (46%) had only been so before their HIV diagnosis, 24 (21%) only after the diagnosis, and 38 (33%) had been pregnant both before and after the HIV diagnosis.

Regarding the 280 episodes of pregnancy, 65% (n = 181) occurred before the HIV diagnosis, and the remaining 99 when the subject was already HIV-positive. Seven of the total pregnancies were achieved though assisted reproductive technology (2.5%).

### Analysis of reproductive desire

Of the 134 women, 49% (n = 66) wanted to have children or more children in the near future. The median age of women with reproductive desire was 32 years (IQR: 28–37) compared to 39.5 years (IQR: 35–42.5) in those with no reproductive desire (p < 0.001). Reproductive desire was more frequent among women of non-Spanish origin than in those of Spanish origin (63% vs. 38%, p = 0.003) (Table 2). No statistically significant differences were found regarding educational level, occupational status or partner and cohabitation status. Reproductive desire was more frequent in women without any children (70% vs. 40%, p = 0.001), but no differences were found by number of pregnancies.

**Table 2:** Univariate and multivariate logistic regression analysis: factors associated with reproductive desire

	**Reproductive desire**					
			**Univariate analysis**		**Multivariate analysis**	
	**N**	**%**	**OR**	**95% CI**	**OR**	**95% CI**
Age, years						
30-39	32/61	52.5	1		1	
<30	23/28	82.1	4.2	1.4-12.3	4.5	1.4-14.3
40-45	11/45	24.4	0.3	0.1-0.7	0.4	0.1-0.9
Country of origin						
Spain	27/72	37.5	1		1	
Others	39/62	62.9	2.8	1.4-5.7	2.2	1.0-5.0
Educational level						
Low	8/15	53.3	1			
Medium	51/103	49.5	0.8	0.3-2.5		
High	7/16	43.7	0.7	0.2-2.8		
Occupational status						
Employed	33/67	49.2	1			
Unemployed	25/47	53.2	1.2	0.6-2.5		
Other situation*	8/20	40.0	0.7	0.2-1.9		
Current partner						
Yes	43/90	47.8	1			
No	23/44	52.3	1.2	0.6-2.5		
Current antiretroviral treatment						
Yes	52/114	45.6	1		1	
No	14/20	70.0	2.8	1.0-7.8	3.6	1.1-12.1
HCV coinfection						
Yes	5/20	25.0	1			
No	61/114	53.5	3.4	1.2-10.1		
Previous children						
Yes	36/91	39.6	1		1	
No	30/43	69.8	3.5	1.6-7.6	3.2	1.3-7.7
Number of pregnancies						
None	10/18	55.6	1			
1-2	37/74	50.0	0.8	0.3-2.2		
> = 3	19/42	45.2	0.7	0.2-2.0		
Information about reproductive health						
Yes	39/66	59.1	1			
No	27/68	39.7	0.4	0.2-0.9		

*Other situation: pensioner, homemaker or student.

With regard to clinical variables, significant differences in reproductive desire were found in women who were coinfected with HCV vs. those who were not coinfected (25% vs. 54% respectively, p = 0.019), among those currently receiving cART vs. those not on cART (46% vs. 70% respectively, p = 0.044), and among women who had been diagnosed at a younger age (<30) vs. medium (30–39) or older age (40–45) (71%, 23% and 6%, respectively, p < 0.001).

In the multivariate analysis (Table 2), the factors associated with reproductive desire were: 1) Age. Considering women between 30 and 39 as the reference category, the OR for women under 30 was 4.5 (95% CI 1.4-14.3), while it was 0.4 (95% CI 0.1-0.9) for women 40 or over; 2) Being from a country other than Spain (OR 2.2, 95% CI 1.0-5.0); 3) Not receiving cART (OR 3.6, 95% CI 1.1-12.1); and 4) Not having any children (OR 3.2, 95% CI 1.3-7.7).

### Reasons for reproductive desire

**Figure 1:**
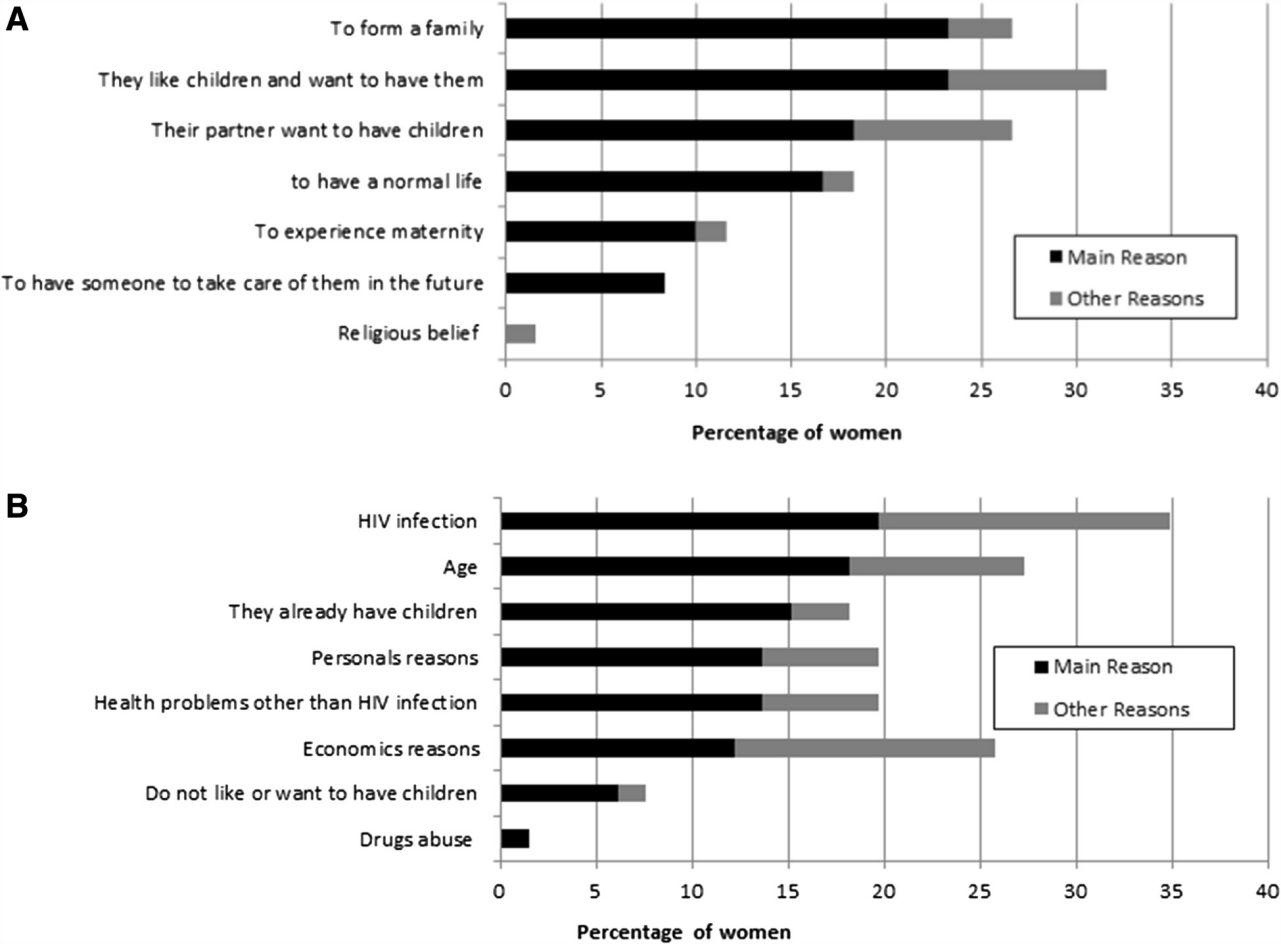
**Responses to reproductive desire: motivations for wanting or not wanting children/more children. A**. Reasons for wanting to have children. **B**. Reasons for not wanting to have children.

Of the 66 women who wanted to have children or more children in the near future, 6 did not state any specific reason or motivation. For the remaining 60 women, the most frequent reasons were *liking children* and *wanting to form a family*, with 23% (n = 14) each. Eighteen percent (n = 11) of these women wanted to procreate because their partner wished to have children of their own, although the women themselves already had children. Fifteen of the 60 women expressed more than one reason for wanting children (Figure 1A).Regarding the 68 women who did not wish to have children or more children in the future, 3 did not express any specific reason or motivation. For the other 66, the main reasons were their HIV infection (20%, n = 13) followed by age (18%, n = 12). About 55% (n = 36) expressed more than one reason for not wanting to have children or more children (Figure 1B).

### Information on reproductive health

Women were asked if they had ever sought or received information about how to have a safe pregnancy since their HIV diagnosis; 49% (n = 66) answered affirmatively. Of them, 59% (n = 39) fulfilled the definition of reproductive desire used in this analysis., i.e., having a desire to be pregnant at present, having unprotected sex with the purpose of having children, or wanting to have children in the near future.

For the majority of these women (82%, n = 54), information was provided by the doctor or other health-care professional involved in the routine clinical follow-up of their HIV infection. Only 6% (n = 4) had received reproductive-health information from non-governmental organizations, and the remaining 12% (n = 8) had looked it up in books, journals or through the internet.

The recommendation to avoid pregnancy had been made to 19% (n = 25) of women at some point since their HIV diagnosis. In 60% (n = 15) of these cases, this recommendation had been made by health-care personnel: in 3 cases, clinical circumstances made it inadvisable to suspend or change antiretroviral treatment; in the rest of the cases, it was related to uncertainty about HIV-infection in the early stages of the epidemic in the 80’s and the 90’s, or had been made by health-care personnel not involved in HIV care, and thus with no specific knowledge about this infection. In 24% (n = 6), the recommendation to not have children came from the family or close relatives and was motivated by fear of HIV.

### Disclosure and social support

Only 10% (n = 14) of interviewed women reported that none of their closest family members knew they were HIV-infected. Of the 120 women who had disclosed their serostatus to their family circle, 33% (n = 40) had told it to all their closest family members, i.e., parents, brothers and sisters, and stable partner, as well as close friends; 25% (n = 30) had only revealed it to their partner; 28% (n = 34) had disclosed it to some family member besides their partner; and 13% (n = 16) had only told it to a very close family member (father, mother, brother, sister, son or daughter).

Overall, 39% (n = 52) reported having experienced discrimination due to HIV infection, with no differences according to disclosure of serostatus. The majority of discrimination situations reported by women occurred in the early moments after their HIV diagnosis. About 37% (n = 19) had experienced discrimination from health-care professionals not used to dealing with HIV-infected patients, in 27% (n = 14) discrimination came from friends or work colleagues, in 15% (n = 8) from a relative, and in 6% (n = 3) from their previous partner. For 15% (n = 8) of women, this discrimination was due to the lack of knowledge and information about HIV in the general population.

Regarding social support evaluated using the 4-item MOS questionnaire, scores were generally high (mean, 76.6, standard deviation (SD): 26.3; median: 87.5). The scores were also high for each of the evaluated dimensions: tangible support (mean 3.71, SD 1.64), emotional/informational support (mean 4.12, SD 1.36), positive social interaction (mean 4.22, SD 1.33), and affectionate support (mean 4.21, SD 1.37).

## Discussion

In our study, we found that half of the women from the Spanish AIDS Research Network Cohort would like to have children in the future and that 35% of their pregnancies were produced after they were diagnosed. The main predictors of women’s reproductive desire were young age, not having children previously, being of foreign origin and not receiving antiretroviral treatment. The reported reasons for wanting to have children were mainly related to the ideas of liking children, wanting to form a family and pleasing their partner. Among the reasons for not wanting children, HIV infection played a major role, but was not a definitive factor [12]. Few of them had disclosed their HIV infection to their social circle and in most cases, only to the partner or very close familiar circle.

No association was found between reproductive desire and HIV disclosure. However, a considerable number of these women had not received information on sexual and reproductive issues, and few of them had consulted or visited fertility clinics.

The proportion of women in our study who wished to be pregnant was much higher than in studies carried out in American [13,14] or African women [15], but similar to what was observed in a Canadian study [16]. In our study, we defined reproductive desire as having a desire to be pregnant at present, or having unprotected sex with the purpose of having children, or wanting to have children in the near future. This means our results may not be fully comparable with the aforementioned studies, which analyzed reproductive desire as a secondary objective within a larger study, with the exception of the article by Loufty et al. [16].

Overall, we observed a high number of pregnancies in these women, both before and after HIV diagnosis; specifically, 35% of pregnancies were produced after the woman was diagnosed with HIV. About 31% of women had been pregnant more than three times. Other studies have observed that the number of HIV-infected women who have consecutive pregnancies has increased, a fact that seems to be associated with age, with not yet having children, and with being diagnosed with HIV after their first pregnancy [17]. The first two reasons are similar to those reported by HIV-negative women for wanting children.

Being younger has been shown to be a predictor of procreation desire in HIV-positive women in all studies [4,5,13-15,18-22]. Women from other countries, mainly from Latin America, have a higher reproductive desire, probably associated with the cultural importance of maternity in these countries [23]. Another factor associated with a higher reproductive desire is not receiving antiretroviral treatment (cART), similar to what was observed by Zhang et al. [24] in a study in Canadian women, where the use of cART was associated with a very low desire for procreation. This result contrasts with the findings of studies in resource-limited settings [13,25,26], where reproductive desire increases when women receive cART, probably as a result of improved health status after beginning treatment. In our setting, where the health system grants universal access to cART, we do not observe such a direct association between receiving cART and reproductive desire, as was also seen in the Canadian study.

Regarding their motivations, we found that even if women already have children from previous relationships, a considerable proportion of them want to have children to please their partners, who wish to have their own children. However, in the multivariate analysis we failed to find any association between reproductive desire and having a stable partner or living with him. Expectations from the family or the partner have a great influence in the procreation desire of persons who live with HIV [14,18,21,27]. As described in the literature, for HIV-infected women, having children can be a key element in feeling useful in society, in doing something meaningful for their partners, and in freeing themselves from the stigma and discrimination experienced by HIV-infected persons [28]. As opposed to other studies focused on African-American women, religious beliefs do not appear to have any influence in the procreation desires of women included in our study [29,30].

Regarding motivations for not having children, in addition to HIV infection, other reasons expressed by women are age and having children already, which are closer to the feelings and concerns of HIV-negative women. In a national survey carried out in 2006 in Spain, the reasons women gave for not wanting more children were that they already had as many as they wanted, that they were too old, that having children was expensive, that children could cause a lot of concern, and for health reasons [31]. In this same survey, the main reasons for wanting children/more children were because it was gratifying (30%), because it was nice to see children grow and develop (20%) and because they enhanced a sense of responsibility and personal development (15%) [31]. In another general population survey conducted in 2003, about women’s attitudes towards children and their upbringing, 52% reported having fewer children than they desired, mainly due to insufficient economic resources (38%) and the difficulty in combining maternity and work (25%). These two reasons were considered even more important when asking respondents why they thought that couples in general had no children or fewer than they desired [32]. Compared to our study population, this survey recruited women who were younger (37% were under 30 years of age), less frequently unemployed (10%), and more frequently had either high or low educational level, and fewer women with medium education.

As previously pointed out, approximately half of the women wanted to have children in the future, form a family and have a normal life, which contrasts with the fact that few of them disclose their HIV infection to their social circle and, in some cases, only the partner, parents or brother or sister knew about it. Our data did not show any association between reproductive desire and HIV disclosure (univariate OR = 0.85; 95% CI 0.27-2.70). Social and family support not only improves the quality of life of persons living with HIV [33,34], but also helps them in making the decision to have children [26,35]. This hypothesis could not be tested in our study, since a reduced version of the MOS questionnaire was considered more suitable for a telephone interview.

The lack of information on reproductive-related issues, both on the part of doctors and other health-care professionals [5] and the women themselves, has important implications for their reproductive-health care. It is essential to include sexual and reproductive health issues in the routine clinical follow-up of women with HIV. Few women consulted or visited fertility clinics (10 of the 134 women interviewed), and few pregnancies were achieved using assisted reproduction techniques. Nevertheless, this proportion is higher than in the general population. In the previously mentioned 2006 survey, the proportion of women who had visited these types of clinics was 1.5% and 3.3%, for women with and without children, respectively [31]. In a Canadian study, the majority of HIV-positive participants reported they would like to have access to information on assisted reproduction treatments, but perceived this as beyond their reach due to the high costs [24]. In our setting, HIV-infected persons can access assisted reproduction treatments within the public health system [36,37]. However, some limitations regarding specific techniques such as sperm washing, and other general criteria such as women’s age or long waiting times, can in the end act as barriers to access.

Although the degree to which our sample is representative of HIV-infected women in Spain, our study is nested within a cohort of patients who visit the recruiting site for the first time and are naïve to antiretroviral therapy, so it is possible that women with a better clinical profile and shorter time since HIV diagnosis are overrepresented in our sample. On the other hand, the method for data collection -the telephone interview- might have deterred the participation of women with a lower educational level, who speak and/or understand Spanish poorly or who have less awareness or are less motivated to participate in research studies. Compared to the population from the municipal registries of women between 15 and 44 years of age, our study had a higher proportion of foreigners, 46% versus 28%, respectively [38]. This is reflected in our results, where we found that being a foreigner is one of the factors associated with reproductive desire. Our sample included a large number of Latin American women, who have a higher birth rate than Spaniards and in whom the desire for maternity is more likely to be influenced by cultural factors related to the traditional role of women in their home countries [19,39].

A limitation of our study is the small sample size analyzed. Almost half of the women selected in the first phase could not be located or were not being followed up when the study was conducted. This was especially true in the largest hospitals (Madrid and Valencia). In these hospitals, which are reference centers for HIV, it is easier for a patient to seek care in the initial phase after diagnosis and then be referred to another center closer to her home. Despite the limited sample, we believe this study provides relevant information to improve reproductive-health services for HIV-infected women. Studies previously carried out in Spain have analyzed the situation of these women once they were pregnant, or have studied changes in mother-to-child transmission over the years [40-42], but they have not evaluated the reasons for wanting or not wanting to have children in the future or the factors associated with this reproductive desire.

Use of telephone interviews to conduct our study offers some advantages: it avoids unnecessary journeys, can be carried out at the most convenient time and place for the person interviewed, and makes it easier for women to answer sensitive questions with greater privacy. At the same time, however, it makes it more difficult to evaluate and study complex subjects like the desire for procreation and social support which could have been approached better in a face-to-face interview. Nevertheless, given the scarcity of studies on this subject in our setting, we think our results can contribute to the understanding of reproductive-health needs of HIV-infected women in Spain.

As noted in the methods section, we used a mixed qualitative-quantitative approach. The qualitative part was constructed by using open-ended questions, which can be difficult to apply in a telephone interview. However, we believe this is the correct approach to elicit motivations underlying reproductive desire, and in the case of social support and disclosure of HIV infection, to explain women’s reasons for wanting or not wanting to have children.

## Conclusion

We can state that the CoRIS HIV-infected women we interviewed have a high reproductive desire, that factors associated with this desire are not fundamentally different from those in the general population of women, and that maternity can even help them to face a situation that they still consider stigmatized and prefer not to disclose. Health-care protocols for handling HIV-positive women should incorporate specific interventions on sexual and reproductive-health, to help them fulfill their procreation desire and experience safe pregnancies.

## Abbreviations

OR: Odds ratios; IQR: Interquartile range; HCV: Hepatitis C virus; HIV: Human immunodeficiency virus; cART: Combined antiretroviral treatment; AIDS: Acquired immunodeficiency syndrome; IDU: Injecting drug user; CI: Confidence interval.

## Competing interests

The authors declare that they have no competing interests.

## Authors’ contributions

VH, BA, and ALL were involved in the study design and participated in the data collection and analysis. VH and ALL wrote the first draft of the manuscript. All authors contributed to data collection, reviewed the draft of the manuscript and approved the final version.

## Pre-publication history

The pre-publication history for this paper can be accessed here:

http://www.biomedcentral.com/1471-2393/14/194/prepub

## Supplementary Material

Additional file 1 Centers and researchers involved in CoRIS. Click here for file

Additional file 2 Questionnaire. Click here for file
