# Supplementary material for: Reproductive desire in women with HIV infection in Spain, associated factors and motivations: a mixed-method study
Source: BMC Pregnancy Childbirth. 2014 Jun 5;14:194. doi: 10.1186/1471-2393-14-194 (PMC4063425; doi:10.1186/1471-2393-14-194)
Supplement: Additional file 1 — Centers and researchers involved in CoRIS. [file 1471-2393-14-194-S1.doc]

**Additional file 1: Centers and researchers involved in CoRIS:**

**Executive committe**: Juan Berenguer, Julia del Amo, Federico García, Félix Gutiérrez, Pablo Labarga, Santiago Moreno y María Ángeles Muñoz.

**Fieldwork, data management and analysis**: Paz Sobrino Vegas, Victoria Hernando Sebastián, Belén Alejos Ferreras, Débora Álvarez, Susana Monge, Inmaculada Jarrín, Adela Castelló.

**BioBanco:** M Ángeles Muñoz-Fernández, Isabel García-Merino, Coral Gómez Rico, Jorge Gallego de la Fuente y Almudena García Torre.

**Participanting centres:**

Hospital General Universitario de Alicante (Alicante): Joaquín Portilla Sogorb, Esperanza Merino de Lucas, Sergio Reus Bañuls, Vicente Boix Martínez, Livia Giner Oncina, Carmen Gadea Pastor, Irene Portilla Tamarit, Patricia Arcaina Toledo.

Hospital Universitario de Canarias (Santa Cruz de Tenerife): Juan Luis Gómez Sirvent, Patricia Rodríguez Fortúnez, María Remedios Alemán Valls, María del Mar Alonso Socas, María Inmaculada Hernández Hernández, Felicitas Díaz-Flores.

Hospital Donostia (San Sebastián): José Antonio Iribarren, Julio Arrizabalaga, María José Aramburu, Xabier Camino, Francisco Rodríguez-Arrondo, Miguel Ángel von Wichmann, Lidia Pascual Tomé, Miguel Ángel Goenaga, Mª Jesús Bustinduy, Harkaitz Azkune Galparsoro

Hospital General Universitario de Elche (Elche): Félix Gutiérrez, Mar Masiá, Cristina López Rodríguez, Sergio Padilla, Andrés Navarro, Fernando Montolio, Catalina Robledano García, Joan Gregori Colomé.

Hospital Universitario La Fe (Valencia): José López Aldeguer, Marino Blanes Juliá, José Lacruz Rodrigo, Miguel Salavert, Marta Montero, Eva Calabuig, Sandra Cuéllar.

Hospital de la Princesa (Madrid): Ignacio de los Santos, Jesús Sanz Sanz, Ana Salas Aparicio, Cristina Sarriá Cepeda.

Hospital San Pedro-CIBIR (Logroño): José Antonio Oteo, José Ramón Blanco, Valvanera Ibarra, Luis Metola, Mercedes Sanz, Laura Pérez-Martínez

Hospital Ramón y Cajal (Madrid): Santiago Moreno, José Luis Casado, Fernando Dronda, Ana Moreno, María Jesús Pérez Elías, Dolores López, Carolina Gutiérrez, Beatriz Hernández, María Pumares, Paloma Martí.

Hospital Reina Sofía (Murcia): Alfredo Cano Sánchez, Enrique Bernal Morell, Ángeles Muñoz Pérez

Centro Sanitario Sandoval (Madrid): Jorge Del Romero Guerrero, Carmen Rodríguez Martín, Teresa Puerta López, Juan Carlos Carrió Montiel, Cristina González, Mar Vera.
