# Supplementary material for: Reproductive desire in women with HIV infection in Spain, associated factors and motivations: a mixed-method study
Source: BMC Pregnancy Childbirth. 2014 Jun 5;14:194. doi: 10.1186/1471-2393-14-194 (PMC4063425; doi:10.1186/1471-2393-14-194)
Supplement: Additional file 2 — Questionnaire. [file 1471-2393-14-194-S2.pdf]

Instituto de Salud Carlos III — Universidad de Alcalá

QUESTIONNAIRE ON REPRODUCTIVE HISTORY AND DESIRE FOR PROCREATION IN WOMEN WITH HIV

A.-SOCIO-DEMOGRAPHIC INFORMATION [A1-A21]

A1a. On what date (approximately) were you diagnosed with HIV? Date: ___/___/____

A1b. Have you ever received antiretroviral treatment for HIV?

YES	1	NO	2	Does not know	9

A2. What is your date of birth? Date: ___/___/____ A2a. Current age: ______ years

A3. In what country were you born?

Spain	1	
Other (specify)	2	

A3a. For how many years have you lived in Spain? _________________ years

A4. About how much do you weigh in Kg., without shoes or clothes?

Weight	Kg

A5. About how tall are you in cm, without shoes?

Height	cm

A6. Have you completed any course of study?

→ **NO** A6a. And which of the following best describes your situation?

**Does not know how to read or write**	1
**Knows how to read and write**	
Has never gone to school	2
Incomplete primary education	3
Does not recall/ No answer	99

→ **YES** **And which of the following best describes your situation?** *(Please specify as precisely as possible, and tell me what course you were in when you finished (or interrupted) your education, as well as the name of that course of study at the time)*

1st LEVEL *(Primary school)* - ***Up to 10 years***	5
2nd LEVEL 1st CYCLE *(Secondary school 1st cycle)* - ***Up to 14 years***	6
2nd LEVEL 2nd *CYCLE (Secondary school 2nd cycle)*- ***Up to 17-18 years***	7
3rd LEVEL *(University diploma / Technical Engineering /* ***3 year degree)***	8
3rd LEVEL *(Bachelor / High Technical Engineering / Doctorate /* ***4-6 year degree)***	9
Other situation: (specify)________________________	10
No answer	99

A7. What is your current employment situation?

Salaried worker	1
Self-employed	2

Retired or pensioner (worked previously)	3	➔**Skip to A14**
Pensioner (did not work previously, housework, etc.)	4	
Unemployed and worked previously	5	
Unemployed and looking for first job	6	
Student	7	
Unpaid domestic work	8	
Other situation	9	
No answer	99	

A8. In what type of business or company do you currently work?

Company ______________________________

Code /___/___/

A9. What is the specific name of your current job? *(Be as precise as possible, for example, "psychiatric nurse", not just nurse").*

Name ______________________________

A10. What do you do in your current job? *(Your primary occupation. e.g., Head of sales department, electronic assembly work.)*

Job ______________________________

Code /___/___/

A11. What type of working day do you have in your current job?

Split shift	1
Continuous workday - mornings	2
Continuous workday – afternoons/evenings	3
Continuous workday - nights	4
Reduced workday	5
Shifts	6
Irregular workday or variable, depending on the day	7
Other type	8

A12. How many hours per week do you currently work, on average?

A13. How many days per week do you work, on average?

Hours per week:-----------h/wk Days per week:--------------d/wk

A14. *(To all)* What is your marital status?

Single	1
Married / Domestic partner	2
Separated /Divorced	3
Widow	4
No answer	99

A15. Do you currently have a partner?

→YES 1 A15a. What is your living situation?

Living with husband / partner	1
Has partner in her city but does not live with him / her	2
Has partner but partner does not live in her city	3

→NO 2

No current partner but has had one or more in the past	4
No current partner and never had one	5
Other (specify)	6

A15b. How many people do you currently live with? __________ persons

A16. Have you ever had complete sexual relations?

Yes	1
No	2
No answer	99

Ask A17 and Skip to ➔ Section I

A17. Do you have any children?

→No

**→Yes**	A17a How many?	/__/__/	What sex?	A17a1.Males	/__/__/	A17a2.Females	/__/__/
	A17b How many adopted?	/__/__/	What sex?	A17b1. Males	/__/__/	A17b2. Females	/__/__/
	A17c How many by Assist. Repro. Tec?	/__/__/	What sex?	A17c1. Males	/__/__/	A17c2. Females	/__/__/

→Women who have not had sexual relations, go to block on DISEASES RELATED TO REPRODUCTION, questions I.

A18. Have you ever been / How many times have you been pregnant, even if the pregnancy was not carried to term? *(If currently pregnant, this pregnancy is also to be included)*

No. of pregnancies *(If "ZERO"* ***DO NOT*** *ask* ***sections on Pregnancies - E and F***

A19. *(Only if currently pregnant)* So you currently are / are not pregnant? (to corroborate)

Current pregnancy YES 1 NO 2 NO ANSWER 9

A20. Are you currently trying to get pregnant?

Yes	1	➔***Skip to Section B***
No	2	
I am not taking any measures, but I do not want to get pregnant	3	

A21. With regard to having children / more children in the near future, tell me: *(Read and circle only one option)*

She would like to have children sometime (does not rule out this possibility)	1
She would like to but cannot have children (either she or partner is infertile)	2
No, does not want to have children / more children	3
Doesn´t know *(do not read)*	4

B. DESIRE FOR PROCREATION [B1-B3]

For women who already have a child/children and would like to have more / For those who do not have any children and would like to have one

B1a. *(For women who have had a child/children).* Why did you have children? *(Open)*

Verbatim: ______________________________

____

B1b. *(To women who want to have (more) children).* Why do you want to have them? *(Open)*

Verbatim: ______________________________

____

B2. *(For women who do NOT want to have children/more children)* Can you tell me the main reason why you do NOT want to have children/another child at present? *(Open)*

Main reason ______________________________

____

B3. *(For women who do NOT want to have children/more children).* Is there any other reason that also influences you negatively? *(Open)*

Others ______________________________

____

C. SEXUAL PARTNERS AND PRACTICES. [C1-C14]

C1. Do you currently have a partner with whom you have sexual relations?

YES	1	
NO	2	Skip to C4
No answer	9	Skip to C4

C2. *(If she has a partner with whom she has sexual relations)* When (approximately) did you begin the (sexual) relation with your current partner?

Date: ___/___/____

C3. *(If she has a partner with whom she has sexual relations)* When was your current partner born?

Date: ___/___/____ (If unknown, write →99) C3a.-Age /___/___/ years

C4. *(To all)* Do you remember at what age (approximately) you had your first sexual relation?

Age /___/___/ years

C5. *(To all)* Can you tell me with how many persons you have had sexual relations in your LIFETIME?

Nº persons/LIFETIME *(If "ZERO" → **error**)*

C6. *(To all)* And in the last 12 MONTHS?

Nº persons/12 months *(If "ZERO" → **Skip to Section D**)*

C7. *(If she has had sexual relations in the last 12 months)* Were any of them a person who has been or is your partner?

Yes	How many? /____/
No	8

C7a. How long has your relationship with your partner lasted? *(To be noted on the following table and for each partner she has had)*

C8. *(If she has had sexual relations in the last 12 months).* That is, can we consider that, in the last 12 months, N (see question C6 and subtract) can be considered casual relations?

Yes	How many?
No casual partners	98

PARTNERS / CASUAL RELATIONSHIPS:

(Frequency of sexual relations with your regular and/or casual partner/s in THE LAST 12 MONTHS. The following questions are a kind of introduction to fill out the table below. The information about casual relationships is collected overall, not for each of the relationships.)

C9. What sex is your regular/casual partner? *Male (M); Female (F)*

C10. Has your regular/casual partner been diagnosed as HIV positive? *Yes (Pos); No (Neg); I don´t know (NR)*

C11. How frequently have you had sexual relations with your regular/casual partner in the last 12 months?
Every day (Daily); Between 1 and 6 times per week (1-6 x/wk); Less than once a week (<1 x/wk); Less than once a month (<1x/m); Never (Never)

C12. When was the last time you had sexual relations with your regular/casual partner? *Approximate date of the relation*

C13.What type of sexual relations do you usually have with your regular/casual partner?
Vaginal intercourse (VI); Vaginal intercourse during menstruation (VIDM); Anal intercourse (AI); Oral sex (OS)

C14. How often to you use condoms in this type of sexual relation with your regular/casual partner?
Never; Occasionally (less than half the time); Frequently (more than half the time); Always

SEE TABLES ON FOLLOWING PAGE

C9-C14. OVERVIEW: PARTNERS IN THE LAST 12 MONTHS

		C9. Sex of partner		C10. Partner's HIV diagnosis			C7a. Duration of relation-ship with partner	C11. Frequency of sexual relations						C12. Date of last sexual relation	C13. Sexual practice / C14. Frequency of CONDOM USE<br>Never (1) Occasional (2) Frequent (3) Always (4)			
		1	2	1	2	9		1	2	3	4	5	6		1	2	3	4
		M	F	POS	NEG	Un-known		Daily	1-6 x/wk	<1x/wk	<1x/m	Never	Only one relation		VI	VIDM	AI	OS
C7	P1																	
	P2																	
	P3																	
	P4																	
	P5																	
	P6																	
C8	Casual part-ners																	

D. CONTRACEPTIVE METHODS [D1-D9]

D1. Before being diagnosed with HIV, what contraceptive method/s did you and/or your partner use? *(Do not read answers. Mark ALL that apply)*

D2. Did either of you change contraceptive method AFTER YOUR diagnosis with HIV?

YES	1	NO	2	→ Skip to D6

D3. What contraceptive method/s did you use in the first 6 months after your HIV diagnosis? *(Fill out the table. Do not read answers. Mark ALL that apply)*

D4. Did you change contraceptive method/s AFTER beginning antiretroviral treatment?

YES	1	NO	2→ Skip to D6	I have never taken antiretroviral treatment	3→ Skip to D6

D5. What type of method/s did you use after beginning antiretroviral treatment? *(Fill out the table. Do not read answers. Mark ALL that apply)*

D6. *(To all)* What type/s of contraceptive method/s have you and/or your partner used in the last 12 months? *(Fill out the table. Do not read answers. Mark ALL that apply)*

	**HIV diagnosis**				**ART**		**Currently**	
	D1. Before		D3. After *(if changed)*		D5. After ART *(if changed)*		D6. Last 12 months	
	**Yes**	**No**	**YES**	**No**	**YES**	**NO**	**YES**	**NO**
a. Male condom	1	2	1	2	1	2	1	2
b. Pill (Oral contraception)	1	2	1	2	1	2	1	2
c. Diaphragm	1	2	1	2	1	2	1	2
d. Intrauterine device (IUD)	1	2	1	2	1	2	1	2
e. Tubal ligation	1	2	1	2	1	2	1	2
f. Vasectomy (partner)	1	2	1	2	1	2	1	2
g. None, I didn´t use any contraceptive methods	1→*to D3*	2	1→*to D5*	2	1	2	1→*to D9*	2
h. I didn´t have (vaginal) sexual relations with men	1→*to D3*	2	1→*to D5*	2	1	2	1→*Next block*	2
i. Others: (Specify)	____________		____________		____________		____________	

D7. *(Only if she has used some method in the last year)* Why did you choose your current contraceptive method/s? What is the main reason? Are there OTHER reasons for your choice?

D7a. Main reason ______________________________

D7b. Others ______________________________

D8. During the past year, have you had unprotected sexual relations (vaginal), even if you did not want to get pregnant?

YES [1] NO [2]

D9. (*ONLY if no method used in the last year)* Why don´t you currently use any contraceptive method/s?

Verbatim ______________________________

E. REPRODUCTIVE HISTORY *(Only for women who have been/are pregnant)* [E1-E113]

E1. On what date (approximately) did your first/second/third... pregnancy end?
(If currently pregnant write CURRENT instead of the date)

E2. So that pregnancy ... was before/after your HIV diagnosis? *Pre HIV; Post HIV*

(***SPECIAL SITUATION:*** *If the date of HIV diagnosis is during the pregnancy, this pregnancy will be considered POST HIV)*

E2a. When in the pregnancy did the diagnosis occur? *1st T; 2nd T; 3rd T; Birth; NR*

E3. Was that pregnancy the result of any assisted reproduction technique? *YES; NO; NR*

E4. How did that pregnancy end? *Live born; Miscarriage; Abortion; Stillborn; Death in the first 7 days; Ectopic pregnancy*

E4a. In the case of abortion, ask the reason why she decided to abort. *(Verbatim)*

(Only for pregnancies occurring AFTER THE HIV DIAGNOSIS.)
E5a. Did you receive ART during that pregnancy? *YES; NO; NR*

E5b. Did you receive ART during labor and/or after the birth? *YES; NO; NR*

E6.-Do you remember what drugs you took:
E6a. Drugs during pregnancy
E6b. Drugs during labor
E6c. Drugs after the birth

(Information about your husband / partner in these pregnancies)
E7. Going back to your first /second ... Was your husband / partner at the time diagnosed with HIV when the pregnancy occurred? *YES; NO; NR*

If the answer is **YES**:

E7a. Do you remember the approximate date when your husband / partner was diagnosed with HIV infection?

E7b. Do you remember if your husband / partner was receiving ART at that time (when the pregnancy occurred)? *YES; NO; NR*

REPEAT THE ABOVE SCHEME FOR EACH PREGNANCY UNTIL TABLE D_1 IS COMPLETED

(Information about your child/children (ALL))

E8. When you were pregnant with your first/second... child, do you remember about how many weeks along you were when the birth occurred, or if the birth was preterm? (Before the 37th week is considered PRETERM) *week number, YES; NO; NR*

E9. Do you remember if it was a vaginal birth, delivery with instruments, programmed Cesarean section or emergency Cesarean section? *Vaginal; Instrumental; Programmed C-section; emergency C-section*

E10. Do you remember if your waters broke spontaneously or were induced? *Spontaneously; Induced*

E11. What was the baby's approximate birthweight?

E12. What was the baby's approximate length?

E13. What is the child's HIV status? *POS; NEG*

E14. Did the baby receive ART at birth? *YES; NO; NR*

E14a. Specify the type of drug:

SEE TABLES ON FOLLOWING PAGE

E1-E7. TABLE E_1 OVERVIEW OF PREGNANCIES

Pregnancy	E1. Date pregnancy ended Month/year	E2 Pre/Post-HIV		E2a. Time of HIV diagnosis in pregnancy	E3.Assist. reproduc. technique	E4. Pregnancy result						E4a. Reasons for abortion	E7.Partner		
		Pre	Post	1T/2T/3T/Birth	YES/NO/NR	Liveborn	Miscarriage	Abortion	Stillborn	Ectopic pregnancy	Death within 7 days	(Transcribe verbatim)	E7. HIV partner	E7a. Date HIV partner	E7b. ART partner
1	/___/____/	1	2			1	2	3	4	5	6		1	/___/____/	1
2	/___/____/	1	2			1	2	3	4	5	6		1	/___/____/	1
3	/___/____/	1	2			1	2	3	4	5	6		1	/___/____/	1
4	/___/____/	1	2			1	2	3	4	5	6		1	/___/____/	1
5	/___/____/	1	2			1	2	3	4	5	6		1	/___/____/	1
	/___/____/	1	2			1	2	3	4	5	6		1	/___/____/	1
	/___/____/	1	2			1	2	3	4	5	6		1	/___/____/	1

- *Put TW if twins and TP if triplets. Use blank lines to complete the information, repeating the order number*

Pregnancy POST HIV (order number)	E5a. ART pregnancy			E5b. ART labor / postpartum			E6a. Specify drugs during pregnancy:	E6b. Specify drugs during labor	E6c. Specify drugs postpartum
	**Yes**	**No**	**NR**	**Yes**	**No**	**NR**	***Specify***	***Specify***	***Specify***
	1	2	9	1	2	9			
	1	2	9	1	2	9			
	1	2	9	1	2	9			

E8-E13. TABLE D_2 OVERVIEW OF DELIVERIES AND CHILDREN BORN

Pregnancy	E8. Preterm birth		E9. Type of delivery				E10. Rupture of membranes		Characteristics of newborn				
			Vaginal	With instruments	Program-med Cesarean-section	Emerging Cesarean-section	Sponta-neous	Induced	E 11. Weight	E12. Length	E13. HIV diagnosis	D14. ART child	D14a. Type of drug
	Week	***YES/NO/NR***	***Circle***				***Circle***		***grams***	***cm***	***POS / NEG***	***YES/NO/NR***	***Specify***
1	/___/___/		1	2	3	4	1	2					
2	/___/___/		1	2	3	4	1	2					
3	/___/___/		1	2	3	4	1	2					
4	/___/___/		1	2	3	4	1	2					
5	/___/___/		1	2	3	4	1	2					

F.-PREGNANCIES POST-HIV AND IDENTIFICATION OF TIME WAITING FOR PREGNANCY (TWP) [F1-F variable]

Situation 1

**FIRST PREGNANCY POST-HIV. NOT CURRENTLY PREGNANT**	**FIRST PREGNANCY POST-HIV AND CURRENTLY PREGNANT**
This pregnancy ended: mo/yr ____/____ (take from previous overview table)<br>Therefore you would have become pregnant in: mo / yr _____ / ______ is that right?	How many weeks pregnant are you?<br>______ weeks<br>Therefore you would have become pregnant in: mo / yr _____ / ______ is that right?

→Begin here IF respondent has had previous pregnancy/ies Pre-HIV:

F1. Had your period come back after your last pregnancy, when you became pregnant again?

Yes	1	No	2	Does not recall	9

F2. Were you breastfeeding when you became pregnant?

Yes	1	No	2	Does not recall	9

→ Begin here if respondent did NOT have a pregnancy pre-HIV:

F3. Did you or your partner use any measure to prevent pregnancy in that period?

YES	1	
NO	2	**➔Skip to F7**
Does not recall	9	

F4. *(If they used birth control)* What type of measure did you use to avoid becoming pregnant? *(Do not read. Mark ALL that apply)* F5. Did you use that birth control method regularly and consistently?

	F4	F5. Regular and consistent use		
Male condom	1	YES	NO	NR
Pill (Oral contraceptives)	2	YES	NO	NR
Diaphragm	3	YES	NO	NR
Intrauterine device (IUD)	4	YES	NO	NR
None, was not using birth control ➔ *skip to F7*	5	YES	NO	NR
Other: (Specify)________________________________	6	YES	NO	NR

F6. *(If they used birth control)* For how long had you been using this/these method/s of birth control when you became pregnant?

Weeks	and/or months	and/or years	NR	999

*➔ Go to situation 2 or Go to next section **G***

F7. *(If they did not use birth control)* For how long had you and your partner not been using any birth control measure/s before you got pregnant?

Weeks	and/or months	and/or years	NR	999

F8. *(If they did not use birth control)* So you hadn´t been using any birth control method/s since about (...)? (to *corroborate, calculate month and year they began to use birth control)*

month ______ and year ______

➔ Go to situation 2 or Go to next section ***G***

Questions F9-F12 For pregnancies achieved using reproductive techniques **(SEE TABLE E_1)**

F9. (*If the pregnancy was achieved through Techniques of Assisted Reproduction) (we know this from the previous Overview Table).* In what clinic did you receive the treatment?

Verbatim ______________________________

F10. Is it Public or Private?

Public	1	Private	2	Does not recall	9

F11. What assisted reproduction procedure did you use to achieve this pregnancy?

Verbatim ______________________________

F12. About when did you begin the assisted reproduction treatments (calculate month and year, to achieve this particular pregnancy)

month ______ and year ______

Situation 2. If CURRENTLY pregnant BUT NOT THE FIRST PREGNANCY after the HIV diagnosis

How many weeks pregnant are you? ______weeks

So you must have gotten pregnant in: month / year _____ / ______ is that right?

F13. Had your period come back after your last pregnancy, when you became pregnant again?

Yes	1	No	2	Does not recall	9

F14. Were you breastfeeding when you became pregnant?

YES	1	No	2	Does not recall	9

F15. Were you or your partner using any type of birth control to avoid pregnancy at that time?

YES	1	
NO	2	➔ **Skip to F19**
Does not recall	9	➔ **Skip to F19**

F16. *(If using birth control)* What type of method did you use to avoid becoming pregnant? *(Do not read. Mark ALL that apply)* F17. Did you use that birth control method regularly and consistently?

	F16	F17. Regularly and consistently		
Male condom	1	YES	NO	NR
Pill (Oral contraception)	3	YES	NO	NR
Diaphragm	4	YES	NO	NR
Intrauterine device (IDU)	5	YES	NO	NR
None, not using birth control ➔ ***skip to F19***	16	YES	NO	NR
Other/s: (Describe)	17	YES	NO	NR

F18. *(If using birth control)* For how long had you been using this/these method/s of birth control when you became pregnant?

Weeks: /___/___/	and/or months /___/___/	and/or years /___/___/	NR	999

➔ *Skip to following section* ***G***

F19. *(If not using birth control)* For how long had you and your partner been having sexual relations without using any birth control measure/s before you got pregnant?

Weeks /___/___/	and/or months /___/___/	and/or years /___/___/	NR	999

F20. *(If not using birth control)* So you had been having relations without using any birth control method/s since about (...)? (To *corroborate, calculate month and year they began to use birth control)*

month and year ____________

➔ *Skip to the next section* ***G***

Questions F21-F24. For pregnancies achieved by assisted reproduction techniques

F21. (*If the pregnancy was achieved by Assisted Reproduction Techniques)* In what clinic did you receive the treatment?

Verbatim ____

F22. Is it Public or Private?

Public [1] Private [2] Does not recall [9]

F23. What assisted reproduction procedure was used to achieve this pregnancy?

Verbatim ____

F24. About when did you being the assisted reproduction treatments?

month ____________ and year ____________

G. HABITS AND OTHER CIRCUMSTANCES DURING TIME WAITING FOR PREGNANCY AND SUCCESSFUL PREGNANCIES [G1-G 17]

Dates: beginning ___/___/_____ end ___/____/_____ TWP1=FIRST PREGNANCY

beginning ___/___/_____ end ___/____/_____ TWPu= CURRENT PREGNANCY (if applicable)

IMPORTANT: All the questions are referred about what the woman did before becoming pregnant

G1. What was your employment situation at the time?

	G1_TWP1	G1_TWPu
The same as now	1	1
Worked outside the home	2	2
Retired or pensioner (worked previously)	3	3
Pensioner (did not work previously, housework, etc...)	4	4
Unemployed and worked previously	5	5
Unemployed and looking for first job	6	6
Student	7	7
Unpaid domestic work	8	8
Other situation	9	9
No answer	99	99

G2. In what type of business or company were you working?

Company		G2_TWP1
Company		G2_TWPu

G3. What was the specific name of your job? *(Be as precise as possible, e.g., "psychiatric nurse", not just "nurse".)*

Name		G3_TWP1
Name		G3_TWPu

G4. What did you do in your job? *(Your main occupation, e.g., head of sales department, electronic assembly worker*

Job		G4_TWP1
Job		G4_TWPu

G5 .What kind of workday did you have at that time in your job?

	G5_TWP1	G5_TWPu
Split shift	1	1
Continuous workdays - mornings	2	2
Continuous workdays – afternoons/evenings	3	3
Continuous workdays - nights	4	4
Reduced workday	5	5
Shifts	6	6
Irregular or variable depending on the day	7	7
Other type *(specify)*________________	8	8

G6. How many hours a week did you work on average? And days of the week?

		G6_TWP1	G6_TWPu
1	Days / week		
2	Hours per week		

G7. At that time, how many times a month did you have sexual relations?

		G7_TWP1	G7_TWPu
	Times per month		
88	Does not want to answer		
99	Does not recall		

G8. Did you use the pill as birth control at any point in the 12 months before that time?

		G8_TWP1	G8_TWPu
1	Yes		
2	No		
9	Does not recall		

G9. How many months before that time did you stop taking the pill?

	G9_TWP1	G9_TWPu
Months		

G10. Were you planning to get pregnant at that time?

	G10_TWP1	G10_TWPu
Yes	1	1
No	2	2
Does not recall	9	9

G11. Did you or your partner seek any kind of help or advice from a physician or other professionals to help you become pregnant?

	G11_TWP1	G11_TWPu
Yes	1	1
No	2	2
Does not recall	9	9

G12. For how long had you been trying to get pregnant when you decided to seek this type of help?

Months	and/or years	G12_TWP1
Months	and/or years	G12_TWPu

G13. Now I am going to ask you some questions about your partner at that time. How old was your partner at that time?

	G13_TWP1	G13_TWPu
Age	/___/___/	/___/___/
Did not have a partner (cases of assisted reproduction technique)	98	98
Does not recall	99	99

G14. What was your partner's employment situation at that time?

	G14_TWP1	G14_TWPu
The same as now	1	1
Worked outside the home	2	2
Retired or pensioner (worked previously)	3	3
Pensioner (did not work previously, housework, etc.)	4	4
Unemployed and worked previously	5	5
Unemployed and looking for first job	6	6
Student	7	7
Unpaid domestic work	8	8
Other situation	9	9
No answer	99	99

G15. What type of workday did your partner have at that time in his job?

	G15_TWP1	G15_TWPu
Split shift	1	1
Continuous workdays - mornings	2	2
Continuous workdays – afternoons/evenings	3	3
Continuous workdays - nights	4	4
Reduced workday	5	5
Shifts	6	6
Irregular or variable depending on the day	7	7
Other	8	8

G16. Did your partner have any disease or health problem at that time that would have prevented or made it difficult for him to be a father (again)?

	G16_TWP1	G16_TWPu
Yes	1	1
No	2	2
Does not recall	9	9

G17. *(Only if he had a disease or problem)* What kind of disease was it?

		G17_TWP1	G17_TWPu
1	Disease	1	1
	G.17a. *Specify*:	_____________	_____________
2	Operation	2	2
	G.17b. *Specify*:	_____________	_____________
3	Difficult or impossible to have sexual relations	3	3
4	Other problems	4	4
	G.17c. *Specify*:	_____________	_____________
9	Does not know	9	9

H. Now I am going to ask some questions about the months in which you became pregnant (ONLY PREGNANCIES POST HIV)[H1-H9]

Collect data for EACH PREGNANCY occurring AFTER the HIV diagnosis.

All questions are referred about factors related to when the woman was pregnant.

H1. Did you work outside the home during the pregnancy? *Yes; No (Months worked)*

H2. How many hours per week on average?

H3. Did you smoke then? *Yes; No; NR*

H4. How much did you smoke? *Number of cigarettes per day*

H5. For each of the following beverages that I am going to name, how much did you drink?

H6. Did you achieve this pregnancy through techniques of assisted reproduction? (to corroborate) *Yes; No; NR*

H7. For how long had you been trying to get pregnant when you decided to seek help using techniques of assisted reproduction? (To corroborate)

ALL THIS INFORMATION IS COLLECTED IN THE FOLLOWING OVERVIEW TABLE

TABLE H

			Pregnancies post HIV				
		measure	*E1*	*E2*	*E3*	*E4*	*E5*
H1	Worked outside home	*Yes(Months)/No*					
H2	Hours/Week		/__/__/	/__/__/	/__/__/	/__/__/	/__/__/
H3	Smoker	*Yes/No*					
H4	Cigarettes	*Cig/day*	/__/__/	/__/__/	/__/__/	/__/__/	/__/__/
H5a	Coffee/tea	*Cups/day*	/__/__/	/__/__/	/__/__/	/__/__/	/__/__/
H5b	Cola soft drinks	*Glasses/day*	/__/__/	/__/__/	/__/__/	/__/__/	/__/__/
H5c	Liquor	*Drinks/day*	/__/__/	/__/__/	/__/__/	/__/__/	/__/__/
H5d	Wine	*Glasses/day*	/__/__/	/__/__/	/__/__/	/__/__/	/__/__/
H5e	Beer	*Glasses/day*	/__/__/	/__/__/	/__/__/	/__/__/	/__/__/
H6	Assist Reprod Tech	*Yes/No/NR*					
H7	Months	*NR:999*	/__/__/	/__/__/	/__/__/	/__/__/	/__/__/

DISEASES RELATED TO REPRODUCTION [I1-I18; J1-J4; K1-K2] (*FOR ALL RESPONDENTS)*

I. Fertility problems of the woman

I1. How old were you when you had your first period?

Age	/__/__/ years
Never had period	98
Does not recall	99

I2. Have you ever had an operation that would make it difficult for you to become pregnant? I2a. When was it?

YES	1	I2a. Year /_________/
NO	2	➔ Skip to I5
Does not recall	9	➔ Skip to I5

I3. *(Only if she has had an operation)* What kind of operation was it?

Tubal ligation (elective)	1
Removal of uterus (hysterectomy)	2
Removal of ovaries	3
Both	4
Other *(specify)*	5
Does not know	9

I4. *(Only if she has had an operation)* If it were possible, would you have liked to have children (more children) after that operation?

Yes	1
No	2
Does not know	9

I4a. *(Only if she has had an operation)* Did you and your partner agree about having / not having more children?

Woman and her partner did NOT agree	1
Woman and her partner DID agree	2
Does not know	9

I5. *(To all)* Have you had any disease or health problem that would prevent or make it difficult for you to become pregnant (again)?

YES	1	
NO	2	→ Skip to I14
Does not recall	9	→ Skip to I14

I6. *(Only if she has had a disease) What type of disease or health problem was it*?

Disease	1	→ I6a. *Specify* :___________________
Difficult or impossible to have sexual relations	2	
Other problems	3	→ I6b.*Specify*:____________________
Does not know	9	

I7. *(Only if she has had a disease)* If it had been possible, would you have liked to have (more) children since then?

Yes	1
No	2
Does not know	9

I8. *(Only if she has had a disease)* Did you and your partner agree?

Woman and her partner did NOT agree	1
Woman and her partner DID agree	2
Does not know	9

I19. *(To all)* Has a doctor ever told you that you have or have had any of the following diseases that I will now read: *(If the woman does not recognize the name, register it as NO)* I10.-(*If she says "YES")* When were you told this for the first time?

I14. Diseases		NO	UNSURE	YES	I10. When told for the first time (month/year)	
I9_1	Pelvic infection/inflammation	2	9	1	I10_1	/_____/________/
I9_2	Fallopian tube infection	2	9	1	I10_2	/_____/________/
I9_3	Ovarian infection	2	9	1	I10_3	/_____/________/
I9_4	Endometriosis	2	9	1	I10_4	/_____/________/
I9_5	Ovarian cysts	2	9	1	I10_5	/_____/________/
I9_6	Perforated appendix	2	9	1	I10_6	/_____/________/
I9_7	Appendicitis	2	9	1	I10_7	/_____/________/
I9_8	Thyroid disease	2	9	1	I10_8	/_____/________/
I9_9	Diabetes	2	9	1	I10_9	/_____/________/

I11. *(To all)* Have you ever used an intrauterine device as a birth control method?

I12 *(Only if she has used an IUD)* For how long? (Months or years)

	I11		
Yes	1	I12. During	Months or years
No	2		
Does not recall	9		

J. Fertility problems of the man

(Only for women who currently have a partner)

J1. Has your husband / partner had any kind of operation to prevent having children (vasectomy)?

J2. (Only if he has had an operation) When was it?

	J1			
YES	1	J2a. In the year /____/	Does not know the year	9999
NO	2	→ Skip to following section **K**		
Does not recall	9			

J3. Has he had any disease or health problem that would prevent or make it difficult for him to become a father (again)?

YES	1	NO	2	Does not recall	9

J4. *(Only if he has had a disease or problem)* What kind of disease or problem was it?

Disease	1	→	J4_1.Specify
Operation	2	→	J4_2. Specify
Difficult or impossible to have sexual relations	3		
Other problems	4	→	J4_4.Specify
Does not know	9		

K. Sexually transmitted diseases (STI)

(To all women interviewed)

K1. Which one/s have you had? and K2. When was it (were they) diagnosed for the first time?

	K1. PREVIOUS				K2. CURRENT		
STI. Diseases	NO	UNSURE	YES	K1a. date (year)	NO	UNSURE	YES
Syphilis	2	8	1	/________/	2	8	1
Vaginitis or cervicitis	2	8	1	/________/	2	8	1
Genital herpes	2	8	1	/________/	2	8	1
Condylomas (genital warts)	2	8	1	/________/	2	8	1
Crabs	2	8	1	/________/	2	8	1
Gonorrhea	2	8	1	/________/	2	8	1
Pelvic inflammatory disease	2	8	1	/________/	2	8	1
HPV infection	2	8	1	/________/	2	8	1
Chlamydia	2	8	1	/________/	2	8	1
Trichomoniasis	2	8	1	/________/	2	8	1
Other *(specify)*	________________			/________/			1

L. KNOWLEDGE OF AND ACCESS TO INFORMATION ABOUT SEXUAL AND REPRODUCTIVE HEALTH [L1-L8]

(To all women interviewed)

L1. Do you have information about how to get pregnant with the least possible risk to yourself and to avoid transmitting the virus to your partner and to the baby?

Yes	1	→L1a. Who provided it? (multiple, note all answers)
No	2	______
Does not recall	9	______

L2. Has anyone ever recommended that you not get pregnant because of your HIV infection? *(open; multiple)*

Yes	1	L2a. Who recommended it?
		L2b. For what reason/s?
No	2	______
Does not recall	9	______

L3. Have you ever gone to or called a Clinic for Assisted Reproduction?

Yes	1	L4. ¿Which one?		
No	2	L4a.	Public	1
Does not recall	9		Private	2

L5. Have you ever undergone any assisted reproduction treatment?

Yes	1	No	2

L6. *(Only if she has undergone an Assisted Reproduction Treatment).* Of the following assisted reproduction procedures, tell me which one/s you have undergone and L7. How many times/cycles? L8. Do you remember in what year you began?

L6. Technique of Assisted Reproduction (TAR)	Yes	No	L7. Cycles	L8. Year
Artificial insemination (AI)	1	2	/___/___/	/_____/
Artificial insemination with sperm washing (HIV+ partner)	1	2	/___/___/	/_____/
Self-insemination (SI)	1	2	/___/___/	/_____/
In vitro fertilization (IVF)	1	2	/___/___/	/_____/
In vitro fertilization with intra-cytoplasmic sperm injection (IVF-ICSI)	1	2	/___/___/	/_____/
IVF-ICSI with sperm washing (HIV+ partner)	1	2	/___/___/	/_____/
Other: *Specify*______________________________	1	2	/___/___/	/_____/

M. SOCIAL AND PARTNER SUPPORT [M1-M7]

(To all women interviewed)

M1. Do those closest to you (parents/children/significant other) know about your HIV infection?

YES [1] NO [2] No answer [9]

M1a. Specify who ____

M2. Do those closest to you at your work/school (work colleagues, fellow students) know about your HIV infection?

YES [1] NO [2] No answer [9]

M3. Have you ever experienced any discrimination, that is, you weren´t allowed to do something, someone bothered you, or made you feel inferior because of your HIV infection?

YES [1] NO [2] No answer [9]

M4.-Please tell me which one/s:
____
____
____
____

M5. *MOSS scale of 4 items to evaluate social support.*

Answer from 1 to 5 where ,…..	Much less than I would like	Less than I would like	Neither a lot nor a little	Almost as much as I would like	As much as I would like
You have someone to help you with daily chores if you fall ill	1	2	3	4	5
You have someone to have fun with (walking, shows, bingo, lunch…)	1	2	3	4	5
You have someone you can go to when you need advice about personal problems	1	2	3	4	5
You have someone to love and who makes you feel wanted	1	2	3	4	5

M6. Support from partner. (*Only if she has a partner)*

	Never	Almost never	Some-times	Almost always	Always
You have the chance to talk about your problems with your partner	1	2	3	4	5
You receive support and understanding from your partner when you need it	1	2	3	4	5
Your partner shows you enough affection	1	2	3	4	5
Your partner shares common responsibilities with you	1	2	3	4	5
Your partner treats you with respect and consideration	1	2	3	4	5

M7. How satisfied are you with your relationship with your partner?

Very satisfied	1
Quite satisfied	2
Not very satisfied	3
Not at all satisfied	4
No answer	9
